# Uncoupling Proteins and Regulated Proton Leak in Mitochondria

**DOI:** 10.3390/ijms23031528

**Published:** 2022-01-28

**Authors:** Afshan Ardalan, Matthew D. Smith, Masoud Jelokhani-Niaraki

**Affiliations:** 1 Department of Chemistry and Biochemistry, Wilfrid Laurier University, Waterloo, ON N2L 3C5, Canada; afshan.ardalan@utoronto.ca; 2 Department of Biology, Wilfrid Laurier University, Waterloo, ON N2L 3C5, Canada; msmith@wlu.ca

**Keywords:** mitochondrial carriers, uncoupling proteins, ADP/ATP carrier, membrane protein structure and function, regulation and mechanism of proton transport, membrane protein oligomerization, ATP synthesis, biphasic proton transport model, alternating access mechanism, reactive oxygen species control

## Abstract

Higher concentration of protons in the mitochondrial intermembrane space compared to the matrix results in an electrochemical potential causing the back flux of protons to the matrix. This proton transport can take place through ATP synthase complex (leading to formation of ATP) or can occur via proton transporters of the mitochondrial carrier superfamily and/or membrane lipids. Some mitochondrial proton transporters, such as uncoupling proteins (UCPs), transport protons as their general regulating function; while others are symporters or antiporters, which use the proton gradient as a driving force to co-transport other substrates across the mitochondrial inner membrane (such as phosphate carrier, a symporter; or aspartate/glutamate transporter, an antiporter). Passage (or leakage) of protons across the inner membrane to matrix from any route other than ATP synthase negatively impacts ATP synthesis. The focus of this review is on regulated proton transport by UCPs. Recent findings on the structure and function of UCPs, and the related research methodologies, are also critically reviewed. Due to structural similarity of members of the mitochondrial carrier superfamily, several of the known structural features are potentially expandable to all members. Overall, this report provides a brief, yet comprehensive, overview of the current knowledge in the field.

## 1. Introduction

Solute carrier proteins (SLCs) are integral Membrane proteins (MP), responsible for transport of solutes across intracellular membranes [1]. The largest family of SLCs in the human body is the mitochondrial carrier superfamily (MCF) or SLC25 with 53 members [2]. MCF proteins are mostly antiporters, transporting different solutes across the inner membrane of mitochondria (IMM) in opposite directions. Some MCF proteins are uniporters, transporting solute molecules in one direction, whereas others are thought to be substrate-proton symporters [2]. Because of amino acid sequence similarities among the MCF members it has been suggested that these proteins have comparable secondary and tertiary structures [3,4]. This review elaborates on recent advances about the structure and function of members of the MCF that are capable of regulated transport of protons across the IMM. We will discuss the common structural and functional features of these proteins, then review the concepts of proton motive force, coupling and uncoupling, and finally discuss the suggested mechanistic models for proton transport.

## 2. Structure of MCF Members

**Figure 1:**
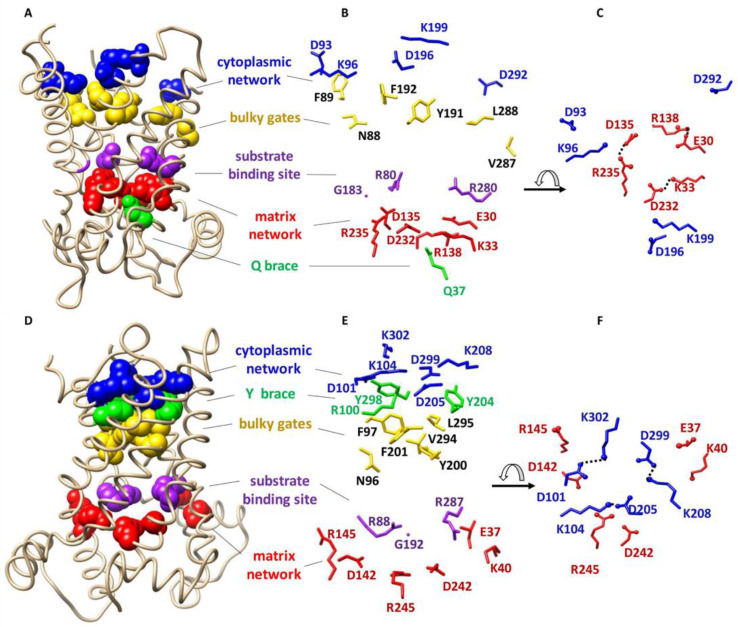
Overall structure and conserved functional residues of AAC. (**A**) X-ray structure of bovine AAC in the cytoplasmic state (1OKC). The conserved functional residues are shown as spheres; (**B**) Detailed representation of the side chains and directions of conserved residues of bovine AAC; (**C**) Top view of broken cytoplasmic and formed matrix networks of bovine AAC in the cytoplasmic state; (**D**) X-ray structure of TtAAC in the matrix state (6GCI). The conserved functional residues are shown as spheres; (**E**) Detailed representation of the side chains of TtAAC; (**F**) Top view of the broken matrix and formed cytoplasmic networks of Tt AAC in the matrix state. Figure generated by USCF Chimera 1.13rc.

Most of the information about the structure of MCF members is based on the knowledge of ADP/ATP carrier protein (AAC) as the most characterized member of the MCF. AAC is the only member of the MCF for which high resolution X-ray structures are available [5,6] (Figure 1A–F). The first high-resolution X-ray structure of this carrier protein (bovine AAC1 or BtAAC) was reported in 2003 (PDB ID: 1OKC) [5]. AAC imports ADP from the intermembrane space (IMS) into the matrix and exports ATP from the matrix to the IMS [6]. The structure of AAC reveals a trilateral pseudosymmetric structure that includes three tandem repeats, that is thought to be typical of all MCF members. Each repeat is composed of ~100 amino acids distributed over two transmembrane (TM) α-helices and a connecting loop containing a hydrophilic short helix parallel to the membrane surface on the matrix side [5] (Figure 1A,D).

Two conserved signature motifs are present at the water-membrane interface of mitochondrial carrier proteins. The first signature motif, PX[DE]XX[RK], is located on odd- numbered helices (helices 1, 3 and 5) close to the matrix side [5,7]. The matrix salt-bridge network (or matrix network) is formed as a result of electrostatic interactions between positive and negative residues of the matrix side signature motifs [5,7] (Figure 1). Conserved glutamine residues, known as the Q-brace/glutamine-brace and located in close proximity to the matrix signature motif (Px[DE]xx[KR]xxxQ) on helices 1 and/or 3 and/or 5, provide extra structural support by forming hydrogen bonds with the salt-bridge network residues 2 (Figure 1). In the past few years computational and experimental evidence have suggested larger (composed of more amino acid residues) and more complex matrix networks for some MCF members, such as AAC1 and uncoupling protein 2 (UCP2) [8]. The second signature motif, [FY][DE]XX[RK], is located on even numbered helices (helices 2, 4 and 6) close to the IMS. Similar to the matrix network, an intramolecular salt-bridge network is also formed from electrostatic interactions of positively and negatively charged residues of the signature motifs close to the IMS, which is known as the cytoplasmic salt-bridge network (cytoplasmic network) [7]. The cytoplasmic network is supported by hydrogen bond formation between tyrosine residues that are in the motif with the residues of the cytoplasmic network (Y brace/tyrosine-brace). MCF members have one to three of each brace (Q or Y) [2] (Figure 1).

Based on the structure of AAC1 it has been proposed that a single conserved common substrate/inhibitor/solute binding site (SBS) is present at the center of the transporter cavity in all MCF members (Figure 1). This binding site resides at the bottom of a solvent accessible cavity and includes three protein-substrate specific contact points at even numbered helices [9,10]. It has been suggested that contact points I (located on helix 2) and II (located on helix 4) can confer substrate specificity to the carrier; however, it seems that the positively charged contact point III (located on helix 6) does not contribute to substrate specificity [2] (Figure 1).

Besides signature motifs, other sequences are also conserved in the members of MCF, such as the πGπXπG motif on helices 1, 3 and 5 and the πXXXπ motif on helices 2, 4 and 6 [6]. The symbol π refers to amino acids with short side chains; however, occasionally amino acids with bulky side chains are found at the π positions 2.

Formation of homo- and/or hetero-oligomers in the membrane to optimize/regulate protein functions is common among MPs [11,12,13]. Therefore, understanding oligomerization and interconversion between different oligomeric states can provide insights about a protein’s mechanism of function [13]. Lipids can influence and control protein oligomerization in the membrane [13]. The role of lipids in controlling oligomerization have been shown to be more important in MPs with weakly interacting subunits and small oligomerization interface(s) (small, buried surface area and no salt-bridge formation between the subunits) [12]. It has also been suggested that cells can alter lipid composition of the membranes to regulate the presence and abundance of oligomeric species [12].

The presence of a GXXXG motif on TM helices has been shown to be involved in homo-oligomerization of MPs as a result of helix-helix interactions [14]. This motif was first shown to play an important role in dimerization of glycophorin A [15]. NMR structure of the glycophorin A showed that two glycine residues, that are separated from one another by three amino acid residues, form a groove that allows the helices to pack closely [16]. Furthermore, the GXXXG motif is known to be the most abundant sequence involved in helix–helix association of E. coli inner MPs (~80%) [14,17]. In addition to GXXXG, πXXXπ (π = small residues such as G, A, S or T) and GXXXAXXG have also been shown to play an important role in oligomerization of proteins [14]. On the other hand, such oligomerization motifs are very abundant in MPs and cannot be used as the only evidence to predict a dimerization interface [14].

The quaternary structure and protein-lipid interactions of members of the MCF are not fully understood and are subjects of debate between researchers [11,18,19,20]. Some reports suggest that all mitochondrial carriers function as monomers [19,21], while others propose oligomeric structures for many of them [11,20]. More specifically, among proton transporters of the MCF, there has not been a consensus on the functional molecular forms of AAC [21,22,23], UCP [11,18,21,24] and phosphate carrier (PIC) [20,21,25]. It has been suggested that AAC forms either a monomer [21] or a dimer [22], or both [26] in the membrane. Out of six molecules of cardiolipin (CL) that are bound to subunits of dimeric AAC, two are thought to sit at the dimer interface and act as a “glue” between the monomeric subunits [22]. Furthermore, it has been suggested that CL might have a role in cross-talk between AAC subunits in the dimer [22]. Moiseeva et al. [26] showed that external factors (such as tonicity of the mitochondria or incubation at 4 °C) can influence the oligomerization of AAC [26]. PICs are also proposed to function either as a monomer [21] or dimer [25]. The case of UCPs is more complicated, since monomeric [27], dimeric [24], and tetrameric [11,28,29] structures have been all reported to be functional.

## 3. Mechanism of Function in MCF Members

**Figure 2:**
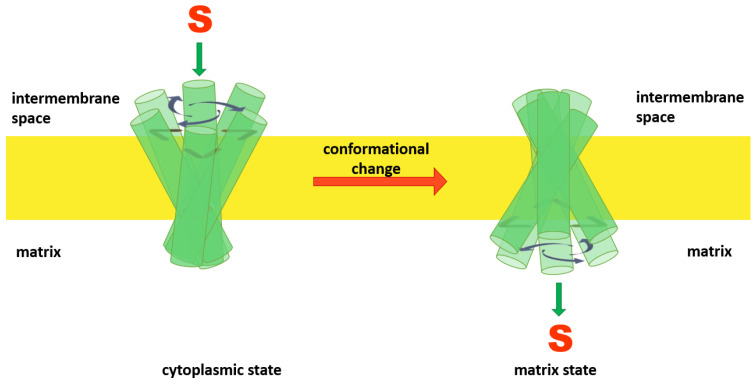
Alternating access mechanism is proposed to be a common mechanism for substrate translocation by members of the MCF. In this model the protein’s cavity opens alternately towards the cytoplasmic and matrix sides alternatively.

Comparable amino acid sequences and the presence of conserved motifs led to suggestions that all members of the MCF transport their respective solutes (substrates) via an alternating access mechanism [2,30]. In this hypothesized mechanism, mitochondrial anion carriers can adopt two different conformational states: a cytoplasmic state (V), in which the solvent-accessible cavity of the carrier is open towards the IMS, the cytoplasmic network is broken and the matrix network is intact (like the letter “V”); and the matrix state (Λ), in which the solvent-accessible cavity of the carrier is open towards the matrix side (like the symbol “Λ”), the cytoplasmic network is formed and the matrix network is broken [9]. The model predicts that members of the MCF can alternate between matrix and cytoplasmic states by movement of the helices towards or away from the central Z axis (normal to the bilayer) as a consequence of substrate binding [6] (Figure 2).

## 4. Proton Motive Force and Coupling

Being the powerhouse of the cell, mitochondria transform energy to produce consumable energy molecules (ATP) that can be used to drive metabolic processes in the cell. For this purpose, the tricarboxylic acid (TCA) cycle [31] (also known as the citric acid cycle or Krebs cycle) and oxidative phosphorylation take place [32] inside the matrix and across the inner membrane (IMM). The final product of lipid, sugar, or amino acid catabolism, acetyl-CoA enters the Krebs cycle when it is combined with oxaloacetate (a four-carbon molecule) in the mitochondria matrix to form a six-carbon citrate molecule. After seven subsequent enzymatic steps, oxaloacetate is regenerated and can receive another molecule of acetyl-CoA [33]. During the Krebs cycle, the two carbons from the acetyl group of acetyl-CoA are released in the form of two CO_2_ molecules, and the extra electrons are used to generate reduced nicotinamide adenine dinucleotide (NADH) and flavin adenine dinucleotide (FADH_2_) that carry the electrons to the electron transport chain (ETC), which is comprised of a series of protein complexes located in the IMM [8].

The movement of electrons from NADH and FADH_2_ through the ETC components results in the efflux of protons from the matrix to the IMS, thus generating an electrochemical gradient across the IMM widely known as the proton motive force [34] that eventually results in formation of ATP through the activity of the ATP synthase complex [34].

The consequence of translocation of protons from the matrix to the IMS in different steps of the ETC is the generation of a ~200 mV electric potential across the IMM [32]. After the Krebs cycle and ETC, the resulting electrochemical gradient across the IMM is transformed into a more readily consumable form of energy for the cell through a “coupling” process. Coupling of the electrochemical gradient to ATP synthesis was first proposed by Peter Mitchell in 1961 [34]. ATP synthase (also known as complex V and F0F1 ATPase) provides a route for protons to flow in accordance with their concentration gradient from the IMS to the matrix. The outcome of this passage is conformational changes in ATP Synthase that result in ATP synthesis from ADP and Pi [8]. The mechanism of ATP synthesis was hypothesized and developed by Paul Boyer and John Walker between 1964 to 1994 [35]. It is estimated that the transport of between two to five protons are needed for the synthesis of one molecule of ATP. However, relating these numbers to the number of required NADH or FADH_2_ is not straightforward as protons can leak across the IMM through routes other than the ATP synthase complex. Three ATP per one NADH or two ATP molecules per one FADH_2_ are common estimations [32].

## 5. Uncoupling in the Mitochondria

Translocation/leak of protons that are pumped into the IMS during the electron transport process back to the matrix by any paths other than the ATP synthase complex results in dissipation of the electrochemical gradient across the IMM, and thus “uncouples” electron transport from ATP synthesis [36]. Proton leak across the IMM is the outcome of two leakage processes: Inducible and basal proton leak [37]. Inducible proton leak is mainly controlled by a specific family within the MCF known as UCPs and can be regulated by fatty acids, superoxides, lipid peroxidation products and adenine nucleotides [38]. Basal proton leak is non-regulated leakage of protons across the IMM, which depends on the fatty acyl composition of the membrane and the presence of mitochondrial carriers that are able to translocate protons [37,38]. Basal proton leak has been extensively discussed in several reviews [37,38]; our focus will be more on inducible and regulated proton leak in mitochondria.

Inducible proton leak in mitochondria can be closely related to the proton transport function of UCPs—proteins that are not universally present in all mitochondria. UCP1 (thermogenin, the prototypic member of the UCP family) was discovered in 1978 by Heaton et al. in hamsters [39]. This protein was purified from brown adipose tissue of rat and hamster mitochondria in high yields by Lin and Klingenberg within two years of its discovery [40]. Almost 20 years after the discovery of UCP1, four other homologues of UCPs were discovered: UCP2 (1997) [41,42], UCP3 (1997) [43,44], UCP4 (1999) [45], and UCP5 (1998) [46]. UCPs have different expression patterns in the human body. UCP1 is mainly found in brown fat adipose tissue [36]. UCP2 is expressed in several tissues such as skeletal muscles, heart, liver, kidney, lung, pancreas, spleen, and macrophages [36]. UCP3 is mostly expressed in brown adipose tissue and muscles [36]. UCPs 4 and 5 are specifically neuronal and mainly expressed in brain tissues [36]. UCPs 2, 4 and 5 are considered neuronal UCPs, as they are predominantly found in the central nervous system (CNS) [28].

Another candidate for contributing to inducible proton leak in mitochondria is AAC. Even though AAC has long been known as a basal proton leaker, it has recently been suggested to be involved in regulated proton transport [47,48]. The next two sections of this review will discuss the regulated proton leakers of the IMM.

## 6. Regulated Proton Transport across the IMM Is Facilitated by Uncoupling Proteins

As mentioned above, UCPs are the only subfamily of the MCF known to be responsible for regulated proton leak across the IMM [38]. Generally, due to low purification yield and intricate preparation steps (such as extraction from membrane, purification in native form, and reconstitution in a bilayer-mimicking environment) analyses of structure and function of MPs have been difficult, cumbersome, and controversial [49]. UCPs are no exception, as there is not universal agreement on UCPs’ tertiary and quaternary structures [11,18,21,24], or their mechanism of function and inhibition [50,51,52,53].

Inconsistencies regarding UCPs’ structure arise from various sources including the organism in which recombinant UCPs are expressed in or purified from, the method of expression, choice of detergent for extraction, as well as the method of purification and reconstitution. As a continuing challenge in obtaining membrane proteins in their native structure and function and regardless of the type of organism used for expression, as long as a single or a mix of detergents is used for isolation of the protein from cell membranes and its reconstitution in lipid membrane models, reproducing an exact native-like structure for proteins would not be possible. This is due to the inherently denaturing character of detergents and the fundamental differences in their physicochemical properties from those of lipids of cell membranes.

**Figure 3:**
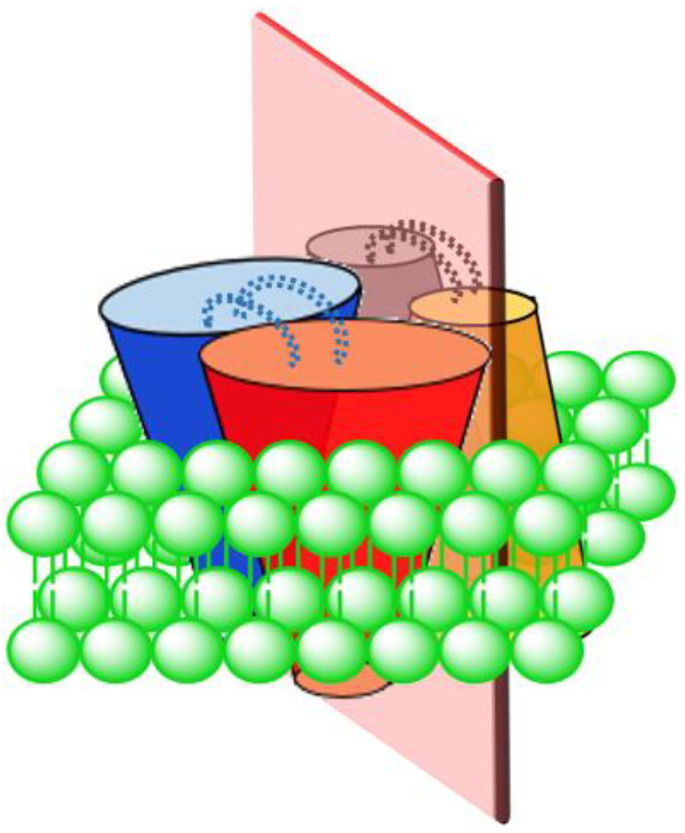
Schematic representation of a pseudosymmetric UCP tetramer. This tetramer is in fact a dimer of dimers with two tight-binding interfaces (between red and blue subunits and between yellow and gray subunits) and a loose-binding interface (shown with a light red plate). There are two salt-bridges between the monomers within each dimer shown with blue and grey dotted lines.

The traditionally understood structure for UCPs, based on methods such as ultracentrifugation [24], cross-linking [18] and nucleotide binding studies [54], is dimeric. In 2013, a tetrameric (dimer of dimers) structure of UCPs was reported for the first time for UCP1 [11]. In this study, UCP1 was directly expressed in and purified from *E. coli* membranes under native conditions and reconstituted in POPC liposomes for structural and functional analyses [11]. Observation of a high molecular weight band at ~132 kDa on polyacrylamide gel (in a semi-native PAGE analysis) as well as evaluations of the protein structure by circular dichroism (CD), ultracentrifugation, and mass spectrometry led Hoang et al. to report a tetrameric structure for UCP1 [11]. Their data showed that gradual addition of the potent denaturing agent SDS to reconstituted tetrameric UCP1 resulted in a gradual decrease in the abundance of the high molecular weight band (at 132 kDa) and corresponding appearance of bands consistent with the size of dimers (~66 kDa) and monomers (~33 kDa) [11]. Disappearance of the tetrameric UCP1 band occurred at lower concentrations of SDS compared to that of dimeric UCP (the kd values for (tetramer)/(dimer) and (dimer)/(monomer) were 1.3 mM and 23 mM, respectively) [11]. Reconstituted UCPs 2, 4 and 5 in POPC (+CL) liposomes have also been reported to be tetrameric showing a behavior similar to UCP1 upon addition of SDS [28]. These observations led to the proposition that the UCP tetramer is in fact a dimer of dimers, in which there is a loose binding interface between the dimers and a tight binding interface between the monomers within each pair of dimers (Figure 3) [11,28]. Consistent with SDS titration data, computational analysis of a UCP2 tetramer demonstrated that the dissociation energy of tetramer to dimer was ~20 kcal/mol lower than dissociation of dimer to monomer (tetramer to dimer: 60 kcal/mol; and dimer to monomer: 80 kcal/mol) [55]. Molecular dynamics (MD) simulations of UCP2 in POPC bilayer, also demonstrated a pseudosymmetrical structure for the tetramer in which the transport state of each dimeric pair open towards one side of the membrane was opposed by the other dimeric pair open on the other side of the membrane (either VVΛΛ or ΛΛVV) [55]. Furthermore, two salt-bridges were observed between the monomers within a dimer while no salt-bridge was observed between the dimers (Figure 3) [55]. Based on the original assumption that protons (or other substrates) were transported through monomeric subunits, these results suggested a possibility of co-existence of all three monomeric, dimeric and tetrameric UCPs as functional forms of the protein(s).

In a detergent-based study using size exclusion chromatography, CD, electrophoretic assays, and isothermal titration calorimetry (ITC), Lee et al. (2015) suggested that the only functional state of UCP1 is monomeric [27]. These researchers performed their structural evaluations of the protein in a non-native environment (detergent in buffer), used reconstituted proteins in detergent and applied an unclear binding model (for stoichiometry) in their ITC experiment, and had an ambiguous interpretation of their data regarding the molecular form of the protein by ignoring the possibility of oligomerization. These shortcomings undermine the certainty of their conclusions.

It is noteworthy that, in addition to the possibility of self-association, UCPs might be able to interact with other members of the MCF, such as AAC, to form heterodimers [56]. In addition, different homologues of UCPs are proposed to be able to interact with one another and form heterodimers, such as UCP2 + UCP3 [57]. It has been also suggested that homo-tetrameric MPs are most probably formed by dimerization of dimers [11,28,58].

**Figure 4:**
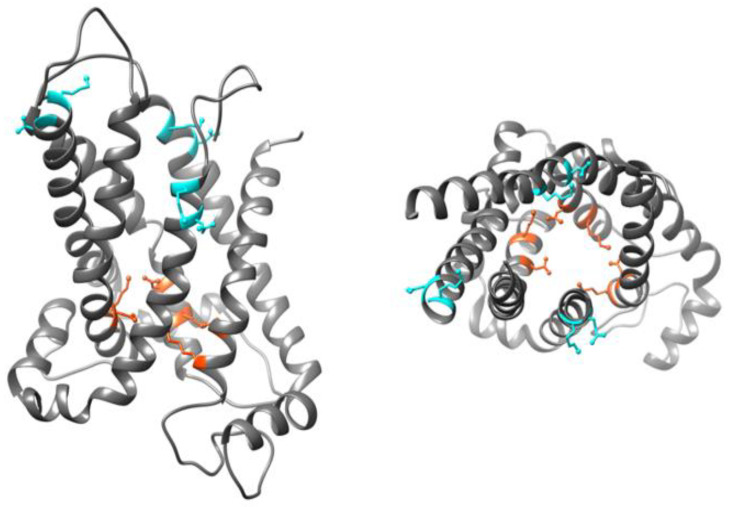
350 ns MD simulated structure of UCP2 in cytoplasmic state. Left: sideview, and right: top view, residues involved in the cytoplasmic and matrix salt-bridge networks are shown in cyan and orange, respectively. Figure was generated by USCF Chimera 1.13rc. Adapted, with modifications, with permission from Ardalan et al. *J. Phys. Chem. B* **2021**, *125*, 9130–9144. Copyright© 2021 American Chemical Society.

Even though there is no available high resolution X-ray structure for UCP homologues, structures of human UCP1 [59] and mouse UCP2 (PDB ID: 2LCK) (Figure 4) [60] have been obtained by NMR spectroscopy. Proteins used for both studies were expressed in and purified from E. coli cells and reconstituted in dodecylphosphocholine (DPC) detergent micelles (UCP1), or detergent (DPC) lipid-mixed micelles (UCP2) for structural analyses [59,60]. Use of DPC detergent micelles as the reconstitution environment has been argued to destabilize UCP structure [49]. The reported NMR structures of UCPs 1 and 2 possess the common structural features of MCF proteins; however, the internal cavity in UCP monomers is larger compared to what is commonly observed in other members of the MCF [49].

It is widely accepted that proton flux by UCPs is activated by fatty acids [28,61]. Evaluation of the effect of fatty acid features (length, structural rigidity, hydrophobicity) on proton transport rate of neuronal UCPs reveals that unsaturated and long chain fatty acids are generally more potent proton transport activators [28,62]. Alterations of circular dichroism (CD) spectra of neuronal UCPs reconstituted in phosphatidylcholine (PC) vesicles upon addition of fatty acids imply that fatty acids are able to induce conformational changes in UCPs [28]. NMR spectroscopy of UCP1 and 2 suggests that there is a groove between helices 1 and 6, which might be a fatty acid binding site; the hydrophobic chain of the fatty acid lays in the groove and its headgroup faces the matrix side [53,59]. Hoang et al. [62] proposed the presence of a specific geometrically optimized binding site for fatty acids in UCPs 2, 4 and 5. Furthermore, mutagenetic and functional analysis of UCPs by different researchers and different methods provide important insights about fatty acid-UCP interactions, as well as residues that are involved in UCP-mediated transport. Table 1 shows the residues that are involved in proton transport regulation along with the major findings of related studies [4,28,53,59,60,62,63,64,65,66,67,68].

**Figure 5:**
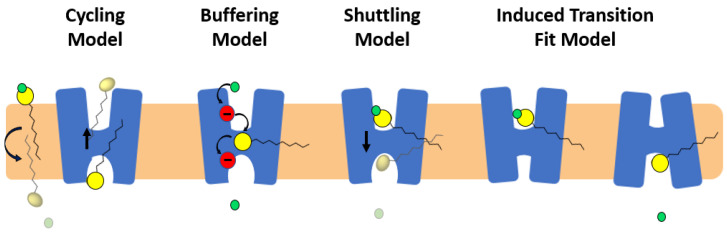
Proposed mechanisms of proton transport in UCPs. In the fatty acid cycling model, protonated fatty acid (yellow head group and a black tail) flip-flops across the lipid bilayer, releases its proton (green circle) and is transported from to the IMS as an anion by UCP. In the buffering model, fatty acid anion binds to UCP and accepts/donates protons via its carboxylate group from and to titratable amino acids of the translocon channel. In the shuttling model long chain fatty acids remain bound to the protein while their head moves back and forth across the IMM (getting protonated in the IMS and releasing the proton into the matrix). In the ITF, UCP changes conformation from cytoplasmic to matrix state and vice versa upon movement of fatty acid across the translocon channel.

Four potential models have been proposed to describe the role of fatty acids in activation of proton transport in monomeric subunits of UCP/UCP1: (i) fatty acid cycling model [51]; (ii) buffering/cofactor model [52]; (iii) proton shuttling model [64] and (iv) alternating access/induced transition fit (ITF) model [63] (Figure 5).

In the fatty acid cycling model, a (protonated) fatty acid that resides in the IMS is transferred to the matrix through a “flip-flop” process across the lipid bilayer and releases its proton to the matrix. Transport of the fatty acid anion back from the matrix to the IMS is then facilitated by UCP (Figure 5) [51]. NMR results from Zhao et al. [59] and MD simulations by Škulj et al. [69] are compatible with the cycling model. In the buffering model, fatty acid anions bind to UCP and accept/donate protons via their carboxylate group from and to titratable amino acids of the translocon channel (the central channel of the carrier), all the way to the matrix side, where the proton is released (Figure 5) [50,52]. The shuttling model proposes that the proton transport mechanism depends on the length of the fatty acid; short-chain protonated fatty acids can be transported from the IMS to the matrix (where they release their proton) via UCPs’ translocon channel, whereas long chain fatty acids remain in the translocation channel. Long-chain fatty acids remain bound to the protein while their head group moves back and forth across the IMM (getting protonated in the IMS and releasing the proton into the matrix) (Figure 5) [64]. In 2017, an updated version of the shuttling model “ITF” was proposed as the fourth proton transport model of UCPs [63]. In this model, the elements of alternating access have been combined with the shuttling mechanism. Based on this ITF model, the arginine residues of the single binding site (R84, R13, R277) of UCP1 attract long chain anionic fatty acids towards the cavity of the protein where D28 is present [63]. Proximity of the anionic fatty acid’s head and D28 will result in increasing the pK_a_ of both fatty acid and aspartate residue leading to attraction of a proton from the IMS. Co-presence of fatty acid and proton in the cavity will lead to further conformational changes from the cytoplasmic to matrix state at which point the proton gets released into the matrix (Figure 5) [63].

All four proposed fatty-acid-mediated transport models involve mechanisms of proton transport through monomeric units of UCPs. Recently, a more complex Biphasic Two-State molecular model has been proposed for proton transport through oligomeric states of UCPs [55,68]. This model is based on a thorough comparative structural and functional analysis of tetrameric UCP2 and several mutants in model lipid bilayers [55,68].

**Figure 6:**
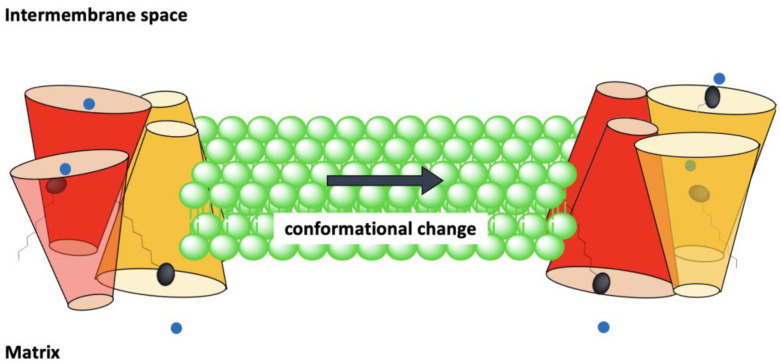
The Biphasic Two-State model for proton transport and inhibition of tetrameric UCP2. The monomeric subunits within a dimeric unit are functional and conformationally correlated. Initially the first dimeric pair (red) is in the cytoplasmic state (open to the IMS) where the fatty acid and proton can be absorbed. Upon movement of the protonated head group of the fatty acid from the IMS to the matrix, the red dimeric pair’s conformation changes to the matrix state (open towards the matrix). The second dimeric pair (yellow), which was initially in the matrix state, transforms to the cytoplasmic state in parallel to the movement of fatty acid’s head group towards the IMS. The four subunits of the tetramer do not have identical conformations at any time; however, the monomers within one dimeric unit are always at comparable conformational states (compare the red subunits conformation with yellow). Deprotonated fatty acids are shown in black, and protons are shown as blue spheres. Fatty acids are protonated at the IMS and deprotonated in the matrix. Changes in conformation of one dimeric pair can induce conformational changes in the other dimeric pair.

In the Biphasic Two-State model each monomer can separately transport protons via the alternating access mechanism. There are two interconnected dimeric units in the tetrameric structure of UCP (tetramer is considered as a dimer of dimers). At any moment, monomers within a dimeric unit are at the same stage/phase of transport. In other words, both monomers of a dimeric unit are either in the cytoplasmic state (VV) or in the matrix state (ΛΛ). There is a binary functional and structural phase difference between the two dimeric units of the tetramer, which means that when one dimeric unit is in the cytoplasmic state and ready to absorb protons in the cytoplasm the other dimeric unit is in the matrix state and release protons into the matrix (pair one: VV, pair 2: ΛΛ, overall tetramer: VVΛΛ). Concurrently, conformational changes of one dimeric pair can influence the active/inactive conformation of the other pair. Considering the loose interface between the two dimeric pairs [55], it has been suggested that dimerization of dimers might be an indication of a higher level of regulation of proton transport by UCPs, as compared with proton transport through monomers [68] (Figure 6). None of these models are universally accepted and continue to be debated [50,69].

It is noteworthy that long-chain fatty acids are not the only activators of proton transport in UCPs; superoxides and alkenals (reactive oxygen-derivatized products), such as HNE (4-hydroxy 2-nonenal), can also activate UCPs [70]. Based on experimental results, it has been suggested that superoxides are activators of proton transport in UCPs from the matrix side [71].

Di- and tri-phosphate purine nucleotides (ADP, ATP, GDP, and GTP) inhibit UCPs’ proton flux activity [72]. The degree of inhibition of UCPs by purine nucleotides is different; for example, UCP2 is more inhibited by ATP compared to ADP [29]. The mechanism of inhibition by purine nucleotides is not fully understood; but the current working model for inhibition is based on mutational studies on UCP1. These studies indicated that three arginine residues are essential in binding of GDP by UCP1: R84, R183, and R277 [65]. Single mutations R183T and R277L resulted in loss of more than 90% of proton transport inhibition without any changes in the uninhibited rate of transport. A single mutation of R88Q caused 99% loss of inhibition, while the proton transport rate doubled [65]. The three R residues are conserved among all human UCPs and make up part of the single SBS (based on homology/sequence alignment with other members of MCF (Table 1)) [9]. It has been demonstrated that addition of purine nucleotides to UCPs reconstituted in lipid vesicles induces minor changes in the protein’s overall conformation [29,73]. One NMR study of UCP2 showed that GDP could broaden NMR resonances on helices 1, 2, 3 and 4 [60], which implies that GDP interacts with these helices [74]. This study suggested that R185 and K141 of UCP2 can interact with GDP [60]. A complementary study by the same group added R88 to the list of GDP-interacting residues [53]. Interestingly, two out of the three amino acids were among those that have been shown to also interact with fatty acids (Table 1) [53]. A suggested inhibitory mechanism of GDP is that it can bind inside the translocation channel of the protein and allosterically displace fatty acid from its binding site [53]. A recent study on the structure and function of tetrameric UCP2 provided evidence that purine nucleotides such as ATP can bind to positive residue at/close to the matrix network (K38, K141, K239, R88, R185, R279) in each subunit and occlude the internal channel thus inhibiting the protein from conformational changes required for proton transport [68].

There are different reports about the stoichiometry of UCP inhibition by purine nucleotides; some have concluded that inhibition is achieved by one nucleotide per monomer [27], while others suggest the stoichiometry is one nucleotide per dimer [75]. The number of nucleotide inhibitors per tetrameric form of UCPs could conform with either suggestion, as only one dimeric unit is in the open state on each side of the membrane (Figure 6).

In addition to protons, all UCP homologues are capable of transporting chloride [29]. However, the rate at which UCPs transport chloride ions is lower compared to that of protons and no fatty acid is required to initiate/activate chloride transport [28,29]. In fact, fatty acids can inhibit the chloride transport activity of UCPs, suggesting that both species share the same path [76].

In addition to protons, UCPs transport a wide spectrum of ions including (but not limited to) monovalent anions such as chloride, bromide, nitrate [62,76], and alkyl sulfonates [77]. It has also been reported that UCP2 is able to transport anionic C4 metabolites such as aspartate, malate, and oxaloacetate [78].

In our experience [11], and as reported elsewhere [22], UCP has no tightly bound CLs; however, the presence of CL in UCPs 2,4 and 5′s lipidic environment affected their proton and chloride transport function and enhanced their helical conformation [29].

## 7. Proton Transport in ADP/ATP Carrier

AAC is an antiporter of ADP and ATP across the IMM. As a result of the conversion between the cytoplasmic and matrix states, AAC is responsible for transporting an amount of ADP and ATP that is approximately equivalent to the average weight of the human body per day [6]. Two inhibitors of AAC, carboxyatractyloside (CATR) [79] and bongkrekic acid (BKA) [80,81], can deactivate the transport and lock the protein structure in cytoplasmic and matrix states, respectively.

The structural elements that are involved in the alternative access mechanism can all be observed in the crystal structures of the carrier (Figure 1) [2,6]: (i) The three salt-bridges of the matrix network (K33-D232, R235-D135 and R235-E30) and the Q-brace (Q37) from BtAAC, V; (ii) The cytoplasmic network salt-bridges (D299-K208, D205-K104 and D101-Q302) and the Y brace Y298, Y204 (and R100) from TtAAC, Λ; and (iii) The SBS residues R88, G192 and R287 for TtAAC and R80, G183 and R 280 for BtAAC.

After the matrix state structure was suggested by Ruprecht et al. (Figure 1) [6], the same researchers proposed a detailed mechanism of transport, which is in agreement with the previously proposed alternating access mechanism [6]. In this mechanism, matrix and cytoplasmic conformational states can be interconverted as a result of interconnected movement of the helices (between 12–17 Å) towards the central z-axis of the carrier or away from it [82]. It has also been proposed that the SBS has minimal movement during the conversion of the two conformational states and is accessible from either side [82]. In fact, the contact points appear to act as a fulcrum during conformational changes [6,82].

A comparison of CATR-locked (V) and BKA-locked (Λ) structures reveals that only 20% of the carrier structure changes during the interconversion of the two conformational states [6]. One notable change is the movement of cytoplasmic ends of the even-numbered helices resulting in formation or disruption of the cytoplasmic network [6]. Surface analyses of the cytoplasmic and matrix states agree with the alternating access mechanism, as in each state the exposed site provides a positively charged accessible surface available for ATP or ADP binding; conversely, the opposite side is closed at the same time with no/very low positively charged surface available for interaction [6].

The proposed transport mechanism of AAC is thorough and detailed [6], however, it suggests that non-solvent accessible gates in both cytoplasmic and matrix states prevent proton leak through the protein. On the other hand, AAC has long been known to cause basal proton leak from the IMS to the matrix [38,83,84]. Furthermore, it has been shown recently that AAC might in fact be involved in regulated proton transport [47,48]. A patch-clamp study of mouse AAC in mitoplasts (mitochondria with the OMM removed) provides valuable insights into this carrier’s proton transport mechanism [47], which is consistent with, yet complementary to, the mechanism proposed by Ruprecht et al. [6]. In this study, AAC was shown to be capable of transporting protons only in the presence of micromolar concentrations of fatty acids on the cytosolic/IMS side [47]. In the proposed mechanism, fatty acid functions as a co-factor bound to the carrier, and its carboxylate head moves through the translocation pathway. Therefore, inhibitors of the translocation pathway (CATR and BKA) inhibit the AAC-mediated proton transport [47]. Fatty acid can bind to the carrier regardless of its initial conformational state (matrix state or cytoplasmic state), and proton transport cannot induce any changes in the carriers’ conformational state [47]. Moreover, translocation of ADP and ATP by AAC decreases the proton transport activity. Based on these observations, it has been hypothesized that AAC functions via two transport modes both sharing the same translocation path: (i) ADP/ATP antiporter mode based on the alternating access mechanism (cytoplasmic state and matrix state interconversions [2]), and (ii) proton transport mode, which relies on presence of/activation by fatty acids [47].

## 8. Importance of Regulated Proton Leak in the Mitochondria

Mitochondria contribute significantly to the production of cellular ROS [85]. Approximately 1 to 2% of the oxygen utilized by mitochondria results in the production of superoxide anion (O2^•−^) [85]. In cases where excess ROS generation leads to oxidative stress, mitochondria overexpress UCPs [86]. The evidence for the preventive role of UCPs against oxidative stress has been discussed in many studies [87,88,89,90]. UCP activity may also decrease the rate of apoptosis (programmed cell death) induced by ROS species [87]. These proteins can be activated by superoxide anions, resulting in the dissipation of the electrochemical gradient across the IMM [85]. Overall, UCPs are considered to contribute to a feedback loop which is activated by ROS, and leads to decrease in superoxide concentration [85,87].

Among UCPs, UCP1 is known to have a thermogenic physiological function in brown adipose tissues. It has also been shown that neuronal UCPs can increase the temperature in neuronal microenvironments during uncoupling activity [86]. As neuronal UCPs mostly accumulate in axon terminals, their thermogenesis can lead to changes in neurotransmission mechanisms [86]. It has been suggested that temperature differences between neuronal cells in the absence and presence of UCPs result in a temperature gradient that facilitates the diffusion of neurochemicals toward their postsynaptic targets [91]. Furthermore, although UCPs decrease the amount of synthesized ATP per mitochondrion, these proteins trigger mitochondrial biogenesis, thereby raising overall cellular ATP levels. Higher cellular ATP concentrations in presynaptic nerve terminals, where UCP is present, assist active processes (such as formation, transportation, and exocytosis of vesicles), and consequently facilitates neurotransmission [86,91].

Mitochondria can modulate the Ca^2+^ content of intact cells via uptake and release mechanisms, which are carried out separately. Mitochondrial ATP production and metabolism can be regulated by Ca^2+^ uptake activity in the IMM, which is mostly performed by the mitochondrial Ca^2+^ uniporter (MCU) [92]. The fundamental role of UCPs in regulating MCU activity has been reported in intact cells and in isolated liver mitochondria [57]. However, the involvement of UCPs in regulation of Ca^2+^ transporters remain controversial [93,94]. Using gene silencing in human and mice cell lines, Trenker et al. [93] showed that UCPs 2 and 3 could affect both the amount and the rate of Ca^2+^ uptake [93]. Additionally, it has been proposed that UCPs can act as conductive ion channels for Ca^2+^ [92,93].

Among members of the MCF, a few are known to transport protons across the IMM (Figure S1). Other than UCPs, of which proton transport is the common regulated function [38], most other transporters of protons are in fact anion carriers, which use the proton (gradient) as a driving force to co-transport other substrates across the IMM [95]. Substrate-proton co-transport by some MCF proteins is accomplished mainly by symport or antiport mechanisms [9,95]. Figure S1 aligns the amino acid sequences and shows the main structural and functional features of these proton transporters.

## 9. Conclusions

Overall, proton leak plays a vital role in mitochondrial bioenergetics as well as ROS generation and oxidative stress. Direct and indirect evidence have shown increased rate of mitochondrial proton leak upon aging [96], Thus proton transporters (basal and regulated) can be considered as potential drug targets for therapy of numerous diseases and understanding the underlying mechanism of proton transport is extremely valuable.

In this report we reviewed the current developments regarding the structure and function of proton transporters with special focus on UCPs. UCPs have been linked to thermogenesis as well as several metabolic diseases such as diabetes, hypertension, obesity, and cancer, making them fascinating targets for drug development [36]. Despite their biological importance there is no agreement on their mechanism of activity or their functional structure. For example, there is evidence that UCPs can exist in the IMM as functional monomers, dimers, tetramers, or a mixture of all. Protons are transported from the internal channel of the monomer or monomeric subunits of the dimer or tetramer. Regardless of what has been observed in experimental models, oligomeric state of UCPs (and other MCF members) might be interchangeable and differ in response to environmental factors or stress (in cell and organism), which can be also observed in mitochondrial structure (morphology) and its dynamic lipid and protein composition. In studying and interpreting dynamic processes in mitochondria or cell, it is essential to always consider the limitations applied to studying the model systems with current technologies when relating the results to a living, independent and ever-changing (non-rigid) system.

## Figures and Tables

**Table 1:** Amino acid residues involved in proton transport regulation.

Protein	H^+^ Transport (Activated by Fatty Acids)	Inhibition (by Purine Nucleotides)	Methods	Findings Related to H^+^ Transport Mechanism	Ref.
UCP2	R279 R60 R241 R88 K141 K16 K271	R185 K141 R88	-NMR -mutagenesis -fluorescence	**1**-The fatty acid chain probably partitions into a groove between helices 1 and 6 which is likely its binding site and the carboxylate head group interacts with R60 and K271. **2**-Fatty acid flipping is required for H^+^ transport activity of UCP2. **3**-GDP can allosterically dislocate fatty acid from its binding.	[53,60]
UCP1	K269 K56		-NMR -mutagenesis -fluorescence -MD simul.	There is a specific binding site between helices 1 and 6 close to the matrix for fatty acid.	[59]
UCP1	D28 R84 R183 R277		-patch clamp of whole mitoplast	**1**-Proposed induced fit mechanism (shuttling model + alternating access) **2**-Long chain low pKa fatty acid anions can inhibit proton transport thus transport of proton and fatty acid is from the same translocation path.	[63,64]
UCP1		R84 R183 R277	-mutagenesis -fluorescence	Single point mutations of R84Q, R183T and R277L resulted in more than 93% decrease in inhibition.	[66]
UCP1	D210 D28 H146 H148	R84 R183 R277	-mutagenesis -fluorescence	E168Q, R84I and R92T inhibited Cl^-^ transport but did not affect proton transport.	[67]
UCP2	R96 k104		-mutagenesis -fluorescence	**1**-Longer fatty acids showed better transport rate: PA > MA > LA **2**-Sensitivity of mutants to fatty acid length was different: K104Q > R96Q > >UCP2 **3**-R76Q and R88Q decreased the chloride transport rate up to 82–83%. **4**-Succinic acid (C4) did not activate proton transport of UCP2.	[62]
UCP2			-fluorescence	Addition of fatty acid inhibited UCP2 inherent chloride transport up to ~50%, suggesting that fatty acids and chloride ions (anions) share the same path.	[28]
UCP2	Salt-bridges: D35-K141 D138-K239 D138-R88 D236-R185 D236-R279	K38 K141 K239 R88 R185 R279	-fluorescence -CD -MD simulation	UCP2 tetramer can transport protons via a **biphasic two-state molecular model:** -All four subunits are functional. -Monomers of each dimer -are in similar transport state. -The function of one dimeric unit could be regulated by the other dimeric unit. ATP can occlude the translocon channel and prevent conformational switching.	[68]
UCP1	Single binding site: D28, R84, R183, S184, I187, S230, R277 (Found based on sequence alignment with AAC, Figure S1)				[9]

## Data Availability

Not applicable.

## Supplementary Materials

The following supporting information can be downloaded at: https://www.mdpi.com/article/10.3390/ijms23031528/s1.
