# Supplementary material for: Uncoupling Proteins and Regulated Proton Leak in Mitochondria
Source: Int J Mol Sci. 2022 Jan 28;23(3):1528. doi: 10.3390/ijms23031528 (PMC8835771; doi:10.3390/ijms23031528)
Supplement: Supplementary file 1 [file ijms-23-01528-s001.zip › ijms-1547519-supplementary.pdf]

Supplementary Materials

Proton Leak in Mitochondria

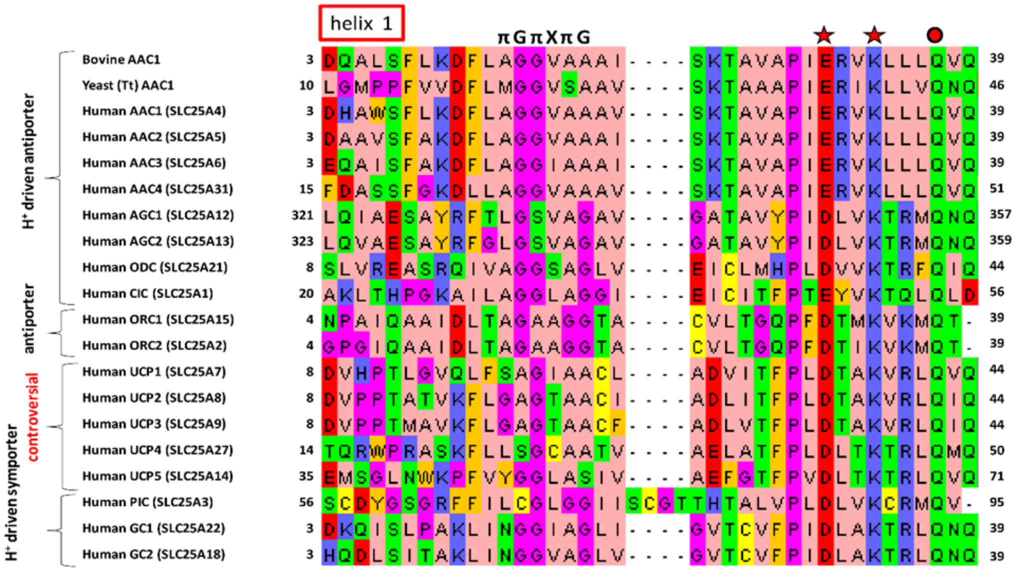

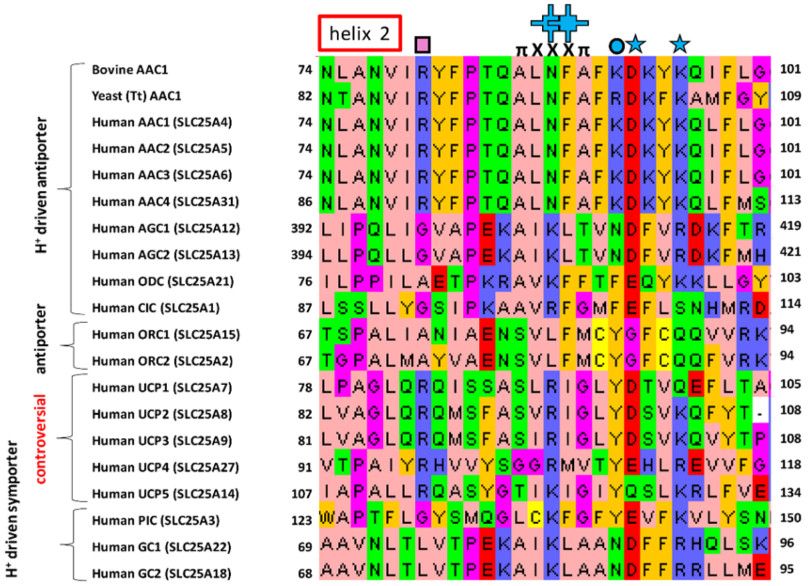

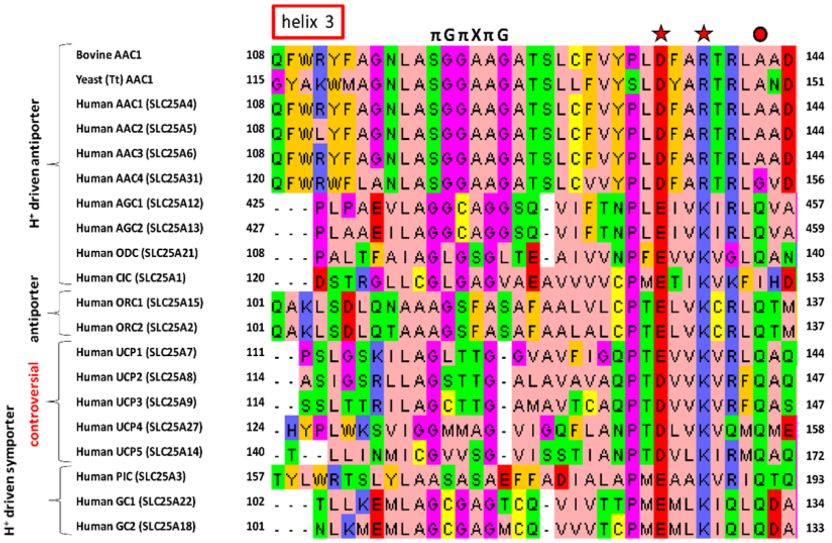
helix 3
πGπXπG
H+ driven antiporter
antiporter
controversial
H+ driven symporter
Bovine AAC1 108 QFWRYFAGNLASGGAAGATSLCFVYPLDFARTRLAAD 144
Yeast (Tt) AAC1 115 GYAKWMAGNLASGGAAGATSLLFVYSLDYARTRLAND 151
Human AAC1 (SLC25A4) 108 QFWRYFAGNLASGGAAGATSLCFVYPLDFARTRLAAD 144
Human AAC2 (SLC25A5) 108 QFWLYFAGNLASGGAAGATSLCFVYPLDFARTRLAAD 144
Human AAC3 (SLC25A6) 108 QFWRYFAGNLASGGAAGATSLCFVYPLDFARTRLAAD 144
Human AAC4 (SLC25A31) 120 QFWRWFLANLASGGAAGATSLCVVYPLDFARTRLGVD 156
Human AGC1 (SLC25A12) 425 ---PLPAEVLAGGCAGGSQ-VIFTNPLEIVKIRLQVA 457
Human AGC2 (SLC25A13) 427 ---PLAAEILAGGCAGGSQ-VIFTNPLEIVKIRLQVA 459
Human ODC (SLC25A21) 108 ---PALTFAIAGLGSGLTE-AIVVNPFEVVKVGLQAN 140
Human CIC (SLC25A1) 120 ---DSTRGLLCGLGAGVAEAVVVVCPMETIKVKFIHD 153
Human ORC1 (SLC25A15) 101 QAKLSDLQNAAAGSFASAFAALVLCPTELVKCRLQTM 137
Human ORC2 (SLC25A2) 101 QAKLSDLQTAAAGSFASAFAALALCPTELVKCRLQTM 137
Human UCP1 (SLC25A7) 111 --PSLGSKILAGLTTG-GVAVFIGQPTEVVKVRLQAQ 144
Human UCP2 (SLC25A8) 114 --ASIGSRLLAGSTTG-ALAVAVAQPTDVVKVRFQAQ 147
Human UCP3 (SLC25A9) 114 --SSLTTRILAGCTTG-AMAVTCAQPTDVVKVRFQAS 147
Human UCP4 (SLC25A27) 124 -HYPLWKSVIGGMMAG-VIGQFLANPTDLVKVQMQME 158
Human UCP5 (SLC25A14) 140 -T--LLINMICGVVSG-VISSTIANPTDVLKIRMQAQ 172
Human PIC (SLC25A3) 157 TYLWRTSLYLAASASAEFFADIALAPMEAAKVRIQTQ 193
Human GC1 (SLC25A22) 102 ---TLLKEMLAGCGAGTCQ-VIVTTPMEMLKIQLQDA 134
Human GC2 (SLC25A18) 101 ---NLKMEMLAGCGAGMCQ-VVVTCPMEMLKIQLQDA 133

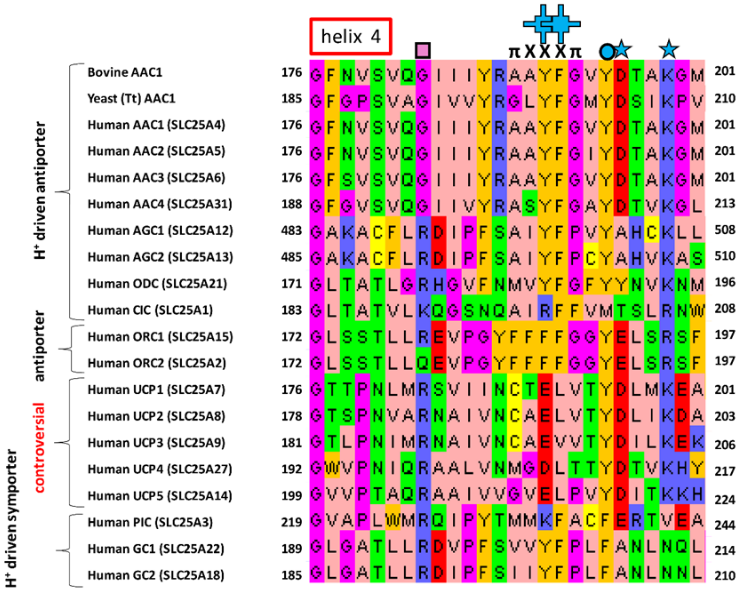
helix 4
πXXXπ
H+ driven antiporter
antiporter
controversial
H+ driven symporter
Bovine AAC1 176 GFNVSVQGIIIYRAAYFGVYDTAKGM 201
Yeast (Tt) AAC1 185 GFGPSVAGIVVYRGLYFGMYDSIKPV 210
Human AAC1 (SLC25A4) 176 GFNVSVQGIIIYRAAYFGVYDTAKGM 201
Human AAC2 (SLC25A5) 176 GFNVSVQGIIIYRAAYFGIYDTAKGM 201
Human AAC3 (SLC25A6) 176 GFSVSVQGIIIYRAAYFGVYDTAKGM 201
Human AAC4 (SLC25A31) 188 GFGVSVQGIIVYRASYFGAYDTVKGL 213
Human AGC1 (SLC25A12) 483 GAKACFLRDIPFSAIYFPVYAHCKLL 508
Human AGC2 (SLC25A13) 485 GAKACFLRDIPFSAIYFPCYAHVKAS 510
Human ODC (SLC25A21) 171 GLTATLGRHGVFNMVYFGFYYNVKNM 196
Human CIC (SLC25A1) 183 GLTATVLKQGSNQAIRFFVMTSLRNW 208
Human ORC1 (SLC25A15) 172 GLSSTLLREVPGYFFFFGGYELSRSF 197
Human ORC2 (SLC25A2) 172 GLSSTLLQEVPGYFFFFGGYELSRSF 197
Human UCP1 (SLC25A7) 176 GTTPNLMRSVIINCTELVTYDLMKEA 201
Human UCP2 (SLC25A8) 178 GTSPNVARNAIVNCAELVTYDLIKDA 203
Human UCP3 (SLC25A9) 181 GTLPNIMRNAIVNCAEVVTYDILKEK 206
Human UCP4 (SLC25A27) 192 GWVPNIQRAALVNMGDLTTYDTVKHY 217
Human UCP5 (SLC25A14) 199 GVVPTAQRAAIVVGVELPVYDITKKH 224
Human PIC (SLC25A3) 219 GVAPLWMRQIPYTMMKFACFERTVEA 244
Human GC1 (SLC25A22) 189 GLGATLLRDVPFSVVYFPLFANLNQL 214
Human GC2 (SLC25A18) 185 GLGATLLRDIPFSIIYFPLFANLNNL 210

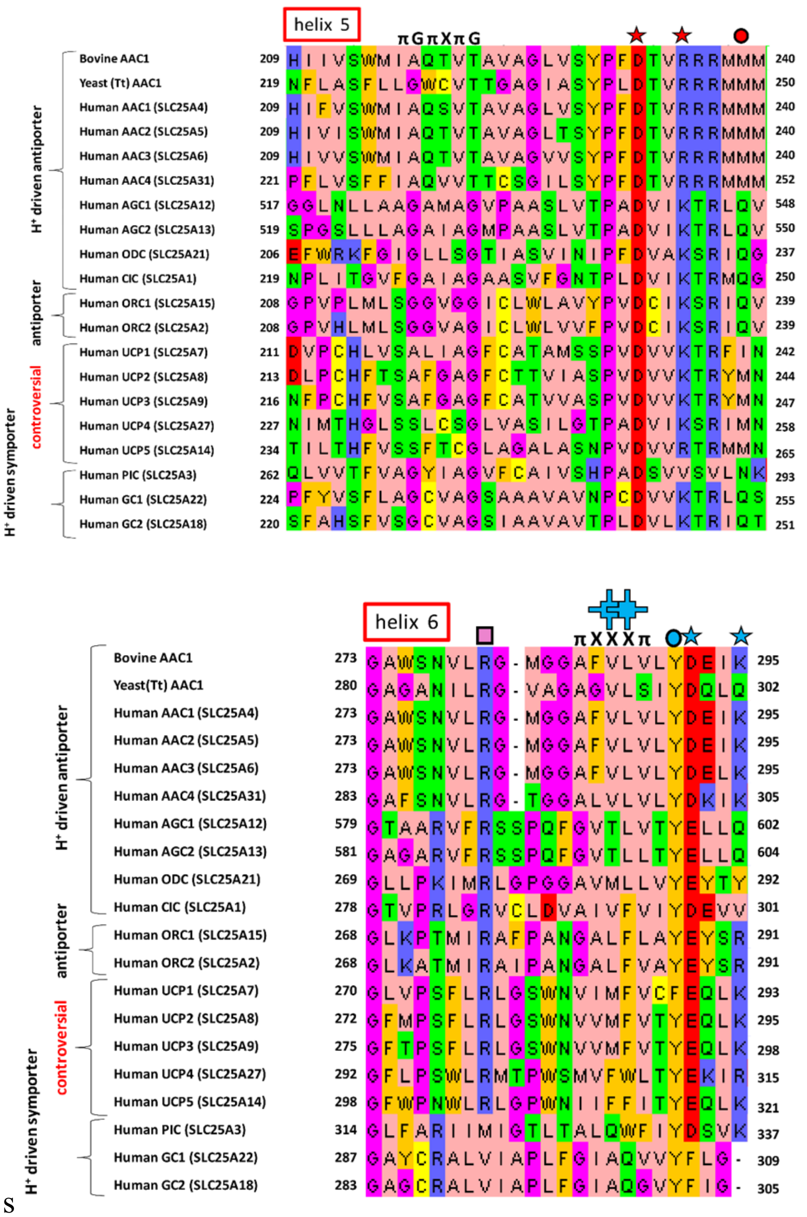

S

Figure S1. Sequence alignment of selected proton transporters of the human MCF, with AAC1 from Bovine and yeast. Alignments were generated using Jalview. Amino acids are color coded as follow: hydrophobic (ILVAM) coral, aromatic (FWY) orange, positive (KHR) blue, negative (DE) red, hydrophilic (STNQ) green, conformationally special (PG) purple and cysteine (C) yellow. Residues involved in the matrix network (red star) and Q-brace (red circle), cytoplasmic network (blue star) and Y-brace (blue circle), bulky residues (blue cross), single binding site (purple square) and the small motifs can be distinguished by their relative signs on top of the columns. AAC: ADP/ATP carrier, AGC: aspartate/glutamate carrier, ODC: oxoadipate carrier, CIC: citrate carrier or tricarboxylate carrier, ORC: ornithine carrier, UCP: uncoupling protein, PIC: phosphate carrier, GC: glutamate carrier. Figure is generated by Jalview 2.11.0.
